# Effects of childhood socioeconomic position on subjective health and health behaviours in adulthood: how much is mediated by adult socioeconomic position?

**DOI:** 10.1186/1471-2458-11-269

**Published:** 2011-04-29

**Authors:** Sarah K Mckenzie, Kristie N Carter, Tony Blakely, Vivienne Ivory

**Affiliations:** 1 Health Inequalities Research Programme, Department of Public Health, University of Otago, Wellington, New Zealand

## Abstract

**Background:**

Adult socioeconomic position (SEP) is one of the most frequently hypothesised indirect pathways between childhood SEP and adult health. However, few studies that explore the indirect associations between childhood SEP and adult health systematically investigate the mediating role of multiple individual measures of adult SEP for different health outcomes. We examine the potential mediating role of individual measures of adult SEP in the associations of childhood SEP with self-rated health, self-reported mental health, current smoking status and binge drinking in adulthood.

**Methods:**

Data came from 10,010 adults aged 25-64 years at Wave 3 of the Survey of Family, Income and Employment in New Zealand. The associations between childhood SEP (assessed using retrospective information on parental occupation) and self-rated health, self-reported psychological distress, current smoking status and binge drinking were determined using logistic regression. Models were adjusted individually for the mediating effects of education, household income, labour market activity and area deprivation.

**Results:**

Respondents from a lower childhood SEP had a greater odds of being a current smoker (OR 1.70 95% CI 1.42-2.03), reporting poorer health (OR 1.82 95% CI 1.39-2.38) or higher psychological distress (OR 1.60 95% CI 1.20-2.14) compared to those from a higher childhood SEP. Two-thirds to three quarters of the association of childhood SEP with current smoking (78%), and psychological distress (66%) and over half the association with poor self-rated health (55%) was explained by educational attainment. Other adult socioeconomic measures had much smaller mediating effects.

**Conclusions:**

This study suggests that the association between childhood SEP and self-rated health, psychological distress and current smoking in adulthood is largely explained through an indirect socioeconomic pathway involving education. However, household income, area deprivation and labour market activity are still likely to be important as they are intermediaries in turn, in the socioeconomic pathway between education and health.

## Background

The influence of childhood and adult socioeconomic position (SEP) on a wider range of adult health outcomes, other than mortality and cardiovascular disease, is becoming a major research focus internationally. There exists three potential mechanisms whereby the socioeconomic environment in childhood can affect health and disease risk in adulthood. Firstly, experience and socioeconomic environments during early life and at subsequent points in the life course may 'accumulate' to influence adult health and disease risk [1]. Secondly, early life socioeconomic circumstances may have a direct effect on adult health through affecting exposure to causal factors acting during 'critical periods of development'. These may have lasting or lifelong effects on the structure or function of organs, tissues and body systems that are not modified in any dramatic way by later life experience [2]. Thirdly, social and economic factors acting in early life may be important, because they influence later life experiences, opportunities and health indirectly via socioeconomic 'pathways' [1,3]. It is the latter that is the focus of this paper.

Adult SEP is considered a pathway or mediating variable because it is heavily influenced by childhood SEP and is itself predictive of subsequent health outcomes. For example, parental SEP can constrain later adult SEP by influencing access to social and economic resources during childhood, especially opportunities for education and other learning experiences [3]. Adult SEP in turn can exert an effect on adult health by determining exposure to causal factors in later life such as income or unemployment. Therefore, it is likely that childhood SEP acts through future adult SEP which in turn influences adult health [3].

Many studies of the effects of childhood SEP on adult mortality [4,5] and cardiovascular disease [6] have shown that adjusting for adult SEP accounts for some or all of the association. In comparison, there are fewer studies to date that have investigated the mediating effects of adult SEP on subjective health measures such as self-reported mental health and specific health behaviours such as smoking and binge drinking in adulthood. Studies that have investigated the effects of adjusting for adult SEP, in the association between childhood SEP and life style risk factors in adulthood such as physical activity, smoking and diet [7-11] and subjective measures such as self-rated health [12,13] and psychosocial functioning, [14,15] show that much if not all of the association of childhood SEP with adult health is explained by adult SEP. However, most of these studies have not directly quantified the proportion mediated and so it is difficult to compare across studies. There is also substantial heterogeneity in the findings of these studies due to a number of reasons as follows.

Firstly, some studies do not present the unadjusted association of childhood SEP and adult health; they mutually adjust for both childhood and adult SEP. As a result how much of the association is mediated by measures of adult SEP cannot be determined [7,16]. Secondly, many studies adjust for only a single measure of adult SEP such as occupational social class [9,10,12,16], employment grade [17] or education [18]. Socioeconomic position is a complex multifaceted construct, [19] therefore such limited scope of adult SEP measures arguably means the underlying construct of adult SEP is not well captured, impairing the assessment of mediation. Thirdly, most studies using more than one measure of adult SEP adjust simultaneously for them all in their modelling, and are therefore unable to assess which individual measures have the greatest mediating effect [15,20-23].

This study takes this research further by assessing the mediating role of adult SEP, on the association of childhood SEP and various adult health measures, by adjusting for a number of adult SEP measures individually (education, household income, labour market activity and area deprivation) as well as together. The aims of this study are to examine a) whether there is an association between childhood SEP and subjective health measures (psychological distress and self-rated health) and health behaviours (smoking and binge drinking) in adulthood and if so b) how much of these associations are mediated by which individual measures of adult SEP.

## Methods

### Data

The data for this analysis came from Wave 3 (2004-2005) of the longitudinal Survey of Family, Income and Employment (SoFIE) *(Wave 1 to 4 version 6). *[24] SoFIE is New Zealand's first national survey designed to study income, family type and employment and how they change over a period of 8 years. It is a nationally representative fixed-panel longitudinal survey of the usually resident population living in private dwellings in New Zealand in 2002. At Wave 3, face-to-face interviews collected information on income levels, employment and education, demographic factors and an array of health questions. Participants were also asked to recall information about their parents' occupation when they were 10 years old. The initial SoFIE sample comprised approximately 11,500 responding private households (response rate of 77%) with 22,165 adults (aged ≥15 years) responding in the first wave of the survey (2002-2003). At Wave 3, 18,950 responding adults answered the health questionnaire (82% of Wave 1 respondents). Of these, 4,090 were excluded as they had no information on parental occupation. The sample was further restricted to working age adults (25 to 64 years old) with complete information on all socioeconomic variables (n = 10,010). Respondents in the final sample were more likely to be aged over 35 years, identify as New Zealand/European, report higher educational qualifications, income and wealth and be less likely to report living in a deprived area compared to the original wave 1 sample. Ethics approval was obtained for the SoFIE Health module from the University of Otago Ethics Committee.

## Measures

### Childhood socioeconomic position

At Wave 3 respondents were asked to recall their parent's occupation when they were aged 10. Occupation was coded using the skills-based New Zealand Standard Classification of Occupations (NZSCO99) [25]. A measure of childhood SEP was derived for each respondent by mapping the highest occupational score (at the two-digit level) of both parents to the New Zealand Socioeconomic Index (NZSEI 1996), a census-derived, occupation based measure of socioeconomic status based on a 'returns to human capital' model of social stratification [26]. NZSEI is a linear scale of ranked occupation, produced using an algorithm involving age, income and education. Thus, variations in occupational orders translate into variations in social stratification and differentiation in lifestyles and life chances. The NZSEI scores in our sample ranged from 20 (representing the occupational group at the lowest) to 65 (the occupational group at the highest ends of the socio-economic hierarchy). NZSEI scores were divided into quartiles creating four discrete childhood socioeconomic groups for descriptive purposes and sensitivity analyses. For regression analyses the NZSEI scores were modelled continuously with the betas scaled by 45 (i.e. the "distance" across the full range of NZSEI scores) and then exponentiated to give odds ratios of the lowest childhood SEP score compared to the highest childhood SEP score.

### Adult socioeconomic position

We used four measures of the respondent's current SEP at Wave 3. Education was measured using the respondent's maximum educational qualification over the three waves and categorised as no qualification, school, post-school vocational, or degree and higher qualifications. Labour market activity was defined as employed, not employed but seeking work, or not employed and not seeking work at the Wave 3 interview date. Equivalised household income (consumer price index adjusted to 2002) was divided into quintiles using the mean household income across the first three waves. The NZ Deprivation (NZDep2001) index provides a measure of small area deprivation which is composed of census variables that reflect aspects of both material and social deprivation [27]. NZDep2001 information was divided into quintiles where NZDepQ1 is the least deprived area and NZDepQ5 is the most deprived.

### Confounders

Sex and age were asked at the initial interview and then checked with the respondents at subsequent waves. At each wave, every adult was also asked their self-identified ethnicity. For analyses we use prioritised ethnicity at Wave 3 where ethnicity was defined as New Zealand/European (those primarily of European descent), Māori (the indigenous people of New Zealand), Pacific (those of Pacific Island descent e.g. Samoan, Cook Island, Fijian), Asian (those of Southeast Asia, China or Indian descent) and other (non NZ/European, non-Māori, non-Pacific and non-Asian) [28].

### Health measures

The global self-rated health question was asked "in general would you say your health is..." on a 5-point Likert Scale: excellent (1), very good (2), good (3), fair (4), poor (5). Self-rated health was dichotomised combining 'fair' and 'poor' to yield a measure of less than good self-rated health, similar to other studies [29]. Non-specific psychological distress was assessed using the Kessler-10 scale (K10) which consists of ten questions on the extent of negative emotional states (mainly anxiety and depressive symptoms) experienced in the four weeks prior to interview [30]. Scores were grouped into four levels according to developed criteria with increasing scores reflecting an increasing degree of psychological distress [31]: low (10-15); moderate (16-21); high (22-29), and; very high distress (≥30) and dichotomised at low/moderate versus high/very high for regression analysis. Current smoking status was derived from information on current and past cigarette use, grouping participants into current smoker or never/ex smoker. Binge drinking was calculated where a respondent had > 1 occasion in the last 4 weeks when they drank 8 (for males) or 6 (for females) standard drinks containing alcohol. Frequency of binge drinking was coded as 0 = never binge drinks, 1 = binge drank monthly, 2 = binge drank 2 times per month, 3 = binge drank weekly, 4 = daily or almost daily binge drinking and dichotomised for logistic regression as binge drinker or not a binge drinker.

### Statistical analyses

First, we examined the association of low childhood SEP compared to high on each of the four health outcomes adjusting for the confounder's age, sex and ethnicity using logistic regression (model 1). Next we adjusted for each adult SEP measure individually (models 2-5) to investigate how much of the association in model 1 is mediated by each SEP measure. Finally, we simultaneously adjusted for all four adult SEP measures (model 6). The observed reduction in the strength of the association between childhood SEP and each health outcome when adjusting for individual adult SEP measures (i.e. models 2 to 5) represents the mediating contribution of that adult SEP measure. The percentage reductions in the excess odds ratios (OR) were calculated as in previous studies [32,33]: (OR_(model 1) _- OR_(adjusted model)_/(OR _(model 1) _-1) × 100%. As a test of whether the changes in strength of the associations upon including intermediaries were statistically significant, we conducted a Hausman test [34]. All data were analysed on unit-level data in the Statistics NZ (SNZ) data laboratory, using SAS 8.2. All tabular numbers of respondents presented in this paper are random rounded to the nearest multiple of five as per SNZ confidentiality protocol.

## Results

The demographic and socioeconomic characteristics of the study sample at Wave 3 by each of the four health outcomes are shown in Table 1. For three of the four health outcomes, the expected relationships were found: adults who were disadvantaged in childhood (as indexed by low parental occupation) were more likely to report poorer health, higher psychological distress and current smoking in adulthood. However, the distribution for binge drinking differed with an even distribution of reported binge drinkers across childhood SEP groups. There were greater proportions of binge drinkers and smokers in the younger age groups. Māori reported the highest proportion of smoking (41.8%) and binge drinking (32.1%).

**Table 1:** Distribution (%) of childhood SEP, demographics and adult socioeconomic characteristics by reports of poor self-rated health, high/very high psychological distress, current smoking and binge drinking in SoFIE respondents aged 25-64 years (n = 10,010)

		Reported less than good health		Reported high/very high psychological distress		Current smoker		Binge drinker	
		Yes (n)	%	Yes (n)	%	Yes (n)	%	Yes (n)	%
Total n	10,010	745	7.4	605	6.0	2140	21.4	2290	22.9
**Childhood SEP**									
I (low)	2575	265	10.3	205	8.0	710	27.6	630	24.5
II	2500	160	6.4	135	5.4	460	18.4	565	22.6
III	2490	185	7.4	140	5.6	515	20.7	530	21.3
IV (high)	2445	140	5.7	125	5.1	455	18.6	565	23.1
**Age (years)**									
25-34	1975	80	4.1	120	6.1	500	25.3	675	34.2
35-44	2965	165	5.6	200	6.7	670	22.6	785	26.5
45-54	2870	250	8.7	160	5.6	580	20.2	550	19.2
55-64	2200	250	11.4	125	5.7	390	17.7	285	13.0
**Sex**									
Female	5440	405	7.4	390	7.2	1135	20.9	880	16.2
Male	4575	345	7.5	215	4.7	1005	22.0	1410	30.8
**Ethnicity**									
NZ European	8185	575	7.0	440	5.4	1590	19.4	1890	23.1
Maori	980	90	9.2	95	9.7	410	41.8	315	32.1
Asian	515	45	8.7	35	6.8	55	10.7	25	4.9
Pacific	325	35	10.8	40	12.3	85	26.2	65	20.0
**Highest educational qualification**									
Degree or higher	1850	65	3.5	55	3.0	160	8.6	330	17.8
Post school vocational	3920	270	6.9	215	5.5	845	21.6	975	24.9
School qualification	2310	150	6.5	140	6.1	505	21.9	575	24.9
No qualification	1930	260	13.5	195	10.1	630	32.6	410	21.2
**Labour market activity**									
Not employed, looking for work	150	20	13.3	20	13.3	60	40.0	35	23.3
Not employed, not looking for work	1675	325	19.4	250	14.9	440	26.3	235	14.0
Working	8185	400	4.9	335	4.1	1640	20.0	2020	24.7
**Household income**									
q1: low - < $21,080	1015	160	15.8	130	12.8	320	31.5	185	18.2
q2: $21,080 - < $34,010	1615	180	11.1	150	9.3	455	28.2	315	19.5
q3: $34,010 - < $49,380	1910	165	8.6	140	7.3	470	24.6	435	22.8
q4: $49,380 - < $72,280	2385	120	5.0	95	4.0	465	19.5	560	23.5
q5: $72,280 - < high	3085	120	3.9	95	3.1	435	14.1	800	25.9
**NZ Deprivation**									
NZDepQ1 (most)	2305	90	3.9	45	2.0	250	10.8	505	21.9
NZDepQ2	2160	125	5.8	110	5.1	335	15.5	450	20.8
NZDepQ3	1840	125	6.8	120	6.5	430	23.4	455	24.7
NZDepQ4	2010	185	9.2	140	7.0	520	25.9	475	23.6
NZDepQ5 (least)	1700	220	12.9	190	11.2	610	35.9	410	24.1

^a ^All numbers of respondents presented in this paper are random rounded to the nearest multiple of five

The distribution of adult socioeconomic indicators by childhood SEP for the study sample is shown in Table 2. Respondents reporting low childhood SEP (group I) were more likely to reside in the most deprived areas (NZDepQ5) in adulthood, report no qualifications, low household income and to be not employed, but looking for work. Childhood SEP was most strongly graded with educational qualifications. For example, the crude OR of no qualifications for low childhood SEP versus high was 3.77, which is higher than similar OR across other adult SEP mediators.

**Table 2:** Distribution (%) of adult socioeconomic indicators by childhood socioeconomic position (SEP) and crude associations (OR) between low versus high childhood SEP and adult SEP in SoFIE respondents aged 25-64 years (n = 10,010)

	Childhood socioeconomic position				
	**I (low)**	**II**	**III**	**IV (high)**	**Crude OR childhood SEP [I v IV]**

	n = 2,575	n = 2,495	n = 2,490	n = 2,450	
**Adult socioeconomic indicators**	col%	col%	col%	col%	OR
**Highest educational qualification**					
Degree or higher	10.5%	13.6%	20.7%	29.6%	0.28
Post school vocational	37.9%	38.5%	42.0%	38.2%	0.99
School qualification	23.1%	25.7%	21.1%	22.4%	1.04
No qualification	28.5%	22.2%	16.3%	9.6%	3.77
**Labour market activity**					
Not employed, looking for work	1.9%	1.2%	1.4%	1.4%	1.37
Not employed, not looking for work	18.3%	16.4%	16.9%	15.3%	1.24
Working	79.8%	82.6%	81.7%	83.1%	0.81
**Household income**					
q1: low - < $21,080	10.9%	11.0%	11.2%	7.3%	1.54
q2: $21,080 - < $34,010	18.8%	16.6%	14.9%	14.5%	1.37
q3: $34,010 - < $49,380	21.2%	18.8%	18.9%	17.1%	1.30
q4: $49,380 - < $72,280	24.7%	23.6%	24.5%	22.4%	1.13
q5: $72,280 - < high	24.5%	30.1%	30.5%	38.6%	0.52
**NZ Deprivation**					
NZDepQ1 (least)	18.6%	22.8%	21.5%	29.2%	0.56
NZDepQ2	19.4%	22.2%	22.9%	21.8%	0.86
NZDepQ3	17.5%	18.0%	19.1%	18.8%	0.92
NZDepQ4	22.3%	20.0%	19.1%	19.0%	1.23
NZDepQ5 (most)	22.1%	16.8%	17.5%	11.2%	2.25

^a ^Pearsons correlation coefficients between childhood SEP and adult socioeconomic indicators were as follows: education 0.18*; household income 0.09*; labour market activity 0.03; NZ Deprivation 0.13* (*p < 0.0001).

Table 3 presents results from the logistic regression models of childhood SEP on adult health outcomes. In model 1 (adjusted for age, sex and ethnicity) low childhood SEP was significantly associated with reporting poorer health (OR = 1.82), high to very high levels of psychological distress (OR = 1.60) and being a current smoker (OR = 1.70). No association was found for binge drinking (and will not be discussed further). When adjusting for each adult SEP measure individually (models 2-5), associations between childhood SEP and adult health were attenuated but remained significant. Adjusting for education attenuated the odds the most towards the null for all health outcomes, leading to a 78% reduction in the odds for smoking, 66% for psychological distress and 55% for poor health. The attenuation following adjustment for household income (model 4) and area deprivation (model 5) was considerably less and labour market activity (model 3) had little effect. Results of the Hausman test for changes in strength of the associations on adjusting for each adult SEP measure were significant for all except labour market activity.

**Table 3:** Odds ratios (OR and 95% CI) for the associations of childhood SEP with poor self-rated health, high/very high psychological distress, current smoking and binge drinking, adjusted for age, sex, ethnicity and individual measures of adult SEP

	Model 1 adjusted for age, sex and ethnicity		Model 2 adjusted for education			Model 3 adjusted for labour market activity			Model 4 adjusted for household income			Model 5 adjusted for NZDep			Model 6 adjusted for all adult SEP		
	**OR**	**95% CI**	**OR**	**95% CI**	**% decline**	**OR**	**95% CI**	**% decline**	**OR**	**95% CI**	**% decline**	**OR**	**95% CI**	**% decline**	**OR**	**95% CI**	**% decline**
**Poor self rated health**	1.82	(1.39-2.38)	1.37	(1.03-1.80)	55%	1.79	(1.36-2.35)	4%	1.55	(1.17-2.03)	33%	1.58	(1.20-2.08)	29%	1.30	(0.98-1.73)	63%
**High/Very high psychological distress**	1.60	(1.20-2.14)	1.20	(0.89-1.62)	66%	1.57	(1.17-2.10)	6%	1.38	(1.03-1.85)	37%	1.37	(1.02-1.84)	38%	1.12	(0.82-1.52)	80%
**Current Smoker**	1.70	(1.42-2.03)	1.15	(0.95-1.39)	78%	1.68	(1.40-2.01)	3%	1.49	(1.24-1.79)	30%	1.49	(1.24-1.79)	30%	1.05	(0.86-1.27)	93%
**Binge drinker**	1.16	(0.98-1.37)															

^a ^Smoking n = 7,424 (ever smokers removed)

^b ^The % decline in the OR compared with model 1 is statistically significant (p < 0.05), using the Hausman test, for all measures except labour market activity

After adjustment for all adult SEP indicators simultaneously (model 6), each association of childhood SEP with health was largely explained (mediated) by adult SEP. For example, the OR for reporting being a current smoker was 1.70, when all adult SEP indicators were added the OR decreased to 1.05. Therefore an estimated 93% of the increased risk of being a current smoker in respondents from a low childhood SEP compared to a high childhood SEP could be attributed to adult SEP in this analysis. Similar reductions were found for psychological distress (80%) and self-rated health (63%). Sensitivity analyses (not shown) using self-rated health and K10 health measures as ordinal variables produced similar results to those obtained from using them as dichotomised measures. When childhood SEP was modelled as quartiles, similar results were produced as when continuous NZSEI scores were used.

## Discussion

The aim of this study was to examine the potential mediating role of multiple individual indicators of adult SEP in the associations of childhood SEP with self-reported health measures and health behaviours. As indicated, few studies to date have investigated the mediating effects of multiple individual measures of adult SEP on subjective health measures such as psychological distress and specific health behaviours such as binge drinking in adulthood. This study highlights the importance of the large mediating effect of education in the link between poor childhood SEP and poor self-rated health, higher psychological distress and current smoking in adulthood. The present associations were explained largely by educational attainment (55% - 78%) whereas household income and area deprivation (indicators of material and social resources) explained less than half of that, and labour market activity virtually none. Our findings support the 'pathways' argument from the life course perspective whereby social and economic factors acting earlier in life may be important because they influence later life experiences, opportunities and health largely through an indirect socioeconomic pathway including education [1,3].

The finding of a much weaker association between childhood SEP and adult health after adjustment for adult SEP (with 95% confidence intervals including the null) is in agreement with studies within other populations [7,8,11,13,14,16,22]. Studies that adjust for only one measure of adult SEP tend to report lesser amounts of mediation, and have adjusted (or indirect) measures of association with 95% confidence intervals excluding the null [10,12]. Using multiple measures of SEP, including education, such as our study will more fully capture the mediation by adult SEP, and hence generate more accurate estimates of the direct and indirect effects of childhood SEP on adult health [35].

Our findings are consistent with previous studies that argue that education is an important explanation for the link between childhood disadvantage and smoking [8,11] and self-rated health in adulthood [13]. Our lack of an association of childhood SEP with binge drinking in adulthood is also consistent with a recent review that found weak and inconsistent evidence to support an association between socioeconomic status in childhood and later alcohol use [36]. Furthermore, there is evidence that adult deprivation is more strongly related to excessive alcohol intake than early life deprivation [20,23].

**Figure 1:**
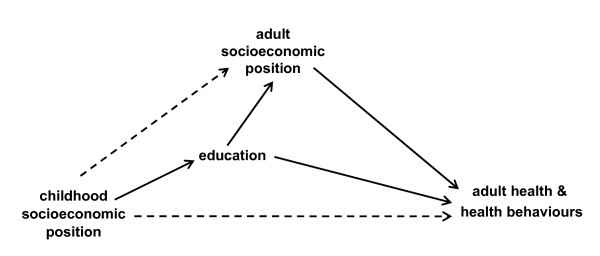
**Simplified model of the associations between childhood socioeconomic position, education, adult socioeconomic position and adult health as conceptualised in this study**.

There are clearly many ways in which childhood disadvantage can affect health many years later, however in this paper we have focused on the socioeconomic pathways. Figure 1, based on our findings, illustrates the possible socioeconomic pathways linking childhood SEP and adult health. Our results suggest that socioeconomic circumstance in childhood has little 'direct effect' on adult health (*dashed line between childhood SEP and adult health in Figure *1) after adjustment for adult SEP.

This however does not imply that socioeconomic circumstances during a person's childhood years are not important determinants of these health outcomes. There is substantial evidence of a powerful effect of family background and childhood disadvantage (assessed by parental education, income, social class and other household characteristics) on educational achievement [37,38]. Education itself may be an important marker of the transition from a SEP largely received from parents to an achieved SEP as an adult. In turn, education shapes health through its impact on socioeconomic circumstance in adulthood since higher levels of education generally are predictive of better jobs, higher incomes, and better housing, neighbourhood and working conditions. These later socioeconomic circumstances in turn impact on health. In other words, some if not most of the effect of childhood SEP will be captured by education, however, other measures of adult SEP are still important as they lie on the pathway from education to health.

### Methodological considerations

One of the key strengths of this study is that we used data from a large nationally representative population in New Zealand with the availability of a wide range of SEP measures and retrospective information on parental occupation. There is however a number of methodological issues that need to be addressed. First, there is the potential for measurement error in the measures of childhood and/or adult SEP. The use of parental occupation at a single age has been argued to be a weak proxy for more complete information on SEP spanning the entire childhood period [39]. However, due to the design of the SoFIE study it was not possible to use prospective repeated measures of childhood SEP over time. Despite the shortcomings of retrospective measures of childhood SEP they do provide a useful opportunity to empirically examine theoretical life course models in the absence of complete data across the life course [40]. If anything, our use of a single measure of childhood SEP may have led to an underestimation of the association between childhood SEP and adult health. Additionally, we have used multiple comprehensive measures of adult SEP to reflect its complex multifactorial nature and to fully account for its mediating effects. We may have therefore more fully adjusted for adult SEP than previous research that has been confined to using a single adult SEP indicator such as occupation or education [9,10,12,16]. This may explain why we found a greater proportion of the effect of childhood SEP on our health measures was mediated by adult SEP than other studies.

It could be argued that measurement error in either our exposure (childhood SEP) or mediating variable (adult SEP) may have biased the relationships found. However, if the parameter of interest is the *proportion *of mediation by adult SEP, and measurement error of both the exposure and mediator are independent and non-differential with respect to the outcome, then the proportion of mediation will be *unchanged *with measurement error of the childhood exposure (although the actual ORs are all reduced) (unpublished work by authors). However, if the mediator (adult SEP) is mismeasured, the proportion due to mediation may be underestimated. Thus, to answer our research question, it is more important to have an accurate assessment of the adult SEP variables than childhood SEP. Of note, and regarding the interpretation of the adult education variable alone, it is almost certain that this variable is misclassified to some extent. If this misclassification is non-differential and independent with respect to both exposure (childhood SEP) and the outcome, our estimate of 55%, 66% and 78% mediation by education probably underestimates the total mediation of the relationship via education.

Second, it is important to note the difficulty with data such as ours to reliably quantify direct and indirect effects due to unmeasured confounding [41,42]. When adjusting for the mediating variable, adult SEP, it may be that there is unmeasured confounding of the adult SEP-health relationship (see Figure 1) [43]. If so, and they are positive confounders, then it may be that we have over-adjusted for adult SEP [41]. However, the strength of the association of these unmeasured confounders with adult SEP and health would have to both be very strong for substantial bias to arise [44,45]. There are also a number of life course measures which can be classed as confounders of the association of childhood SEP with adult health (e.g. childhood health) and between childhood SEP and adult SEP (e.g. genetic inheritance, family environment). The absence of this information in our study may have led to some bias, probably in the direction of an initial overestimation of the total association of childhood SEP with health.

Third, a possible limitation of our analysis is that it does not take into account the possible reverse causation of adult health on adult SEP, where those with pre-existing poor health drift down the social scale i.e. health selection. However, education (which explained the largest proportion of mediation in our analyses) is arguably less likely to be subject to health selection bias as most formal education is complete by young adulthood. Finally, this analysis was restricted to survey respondents who had full exposure and outcome data at Wave 3. However, for selection bias to arise would require the association of childhood SEP, adult SEP and adult health to differ among those respondents included in the final analyses compared to those excluded. This could potentially attenuate the associations found but we do not have any evidence whether this is the case or not.

## Conclusions

Our results suggest that educational attainment is the likely key gateway to socioeconomic trajectories that link childhood SEP and poor adult health, psychological distress and current smoking. Educational attainment is influenced by childhood socioeconomic circumstances and in turn, shapes health through its impact on socioeconomic circumstance in adulthood through better jobs, higher household incomes, and better housing. It is these later socioeconomic circumstances which in turn impact on health. Education might also affect a person's receptivity to health education messages which could have a beneficial influence on their health through health promoting behaviours and lifestyles. This may be especially important in younger people during critical periods when health behaviours such as smoking and drinking patterns are established. Thus, ensuring good access to and quality of education especially for younger people may be the best approach to tackle the transmission of social and economic disadvantage over the life course.

## Competing interests

The authors declare that they have no competing interests.

## Authors' contributions

SM wrote the first draft of the manuscript and carried out the data analyses. SM, KC and TB were responsible for the conception, design and interpretation of the data; VI contributed to the interpretation of the data and revision of the manuscript. All authors commented extensively on subsequent revisions and have read and approved the final manuscript.

## Pre-publication history

The pre-publication history for this paper can be accessed here:

http://www.biomedcentral.com/1471-2458/11/269/prepub
